# Body mass index, type 2 diabetes, and left ventricular function

**DOI:** 10.1186/s12933-017-0649-9

**Published:** 2018-01-04

**Authors:** Katrine Dina Musaeus, Manan Pareek

**Affiliations:** 1 Faculty of Health and Medical Sciences, University of Copenhagen, Blegdamsvej 3B, 2200 Copenhagen N, Denmark; 2 Brigham and Women’s Hospital Heart & Vascular Center, Harvard Medical School, 75 Francis St, Boston, MA 02115 USA; 3 Cardiovascular and Metabolic Preventive Clinic, Department of Endocrinology, Centre for Individualized Medicine in Arterial Diseases (CIMA), Odense University Hospital, Sdr. Boulevard 29, 5000 Odense C, Denmark

**Keywords:** Body mass index, Diabetes mellitus, type 2, Ventricular dysfunction, left, Ventricular function, left

## Abstract

A recent study found that among individuals with a preserved left ventricular ejection fraction ≥ 55%, global longitudinal strain was significantly lower in overweight patients (i.e., body mass index ≥ 25 kg/m^2^) with, but not in those without, type 2 diabetes mellitus. These results contrast previous observations of body mass index as a significant predictor of incident diastolic dysfunction and increased left ventricular mass index among subjects without prevalent diabetes. We discuss potential explanations for the observed discrepancies and general difficulties associated with cardiovascular risk assessment based on body mass index and related metabolic factors.

## To the Editor

We have read with great interest the study by Suto et al. on how overweight might affect left ventricular (LV) function in subjects with and without type 2 diabetes mellitus (T2DM) [1]. The authors evaluated 145 patients with T2DM and 90 healthy controls, matched on age, sex, and LV ejection fraction (LVEF). All participants had a preserved LVEF (≥ 55%). The main finding was that global longitudinal strain (GLS) was significantly lower among overweight patients (i.e., body mass index (BMI) ≥ 25 kg/m^2^) with T2DM, but not among those who were overweight without T2DM. It was suggested that the development of heart failure with preserved EF may be prevented by controlling overweight in patients with T2DM. We believe that this study warrants further discussion.

The results contrast previous observations of BMI as a significant predictor of incident diastolic dysfunction and increased LV mass index among subjects without diabetes at baseline [2]. Although GLS cannot be directly compared with traditional Doppler markers of diastolic function, one possible explanation for the discrepant findings may lie in the suboptimal utilization of BMI data in both studies. Indeed, given its J- or U-shaped association with clinical endpoints, BMI is too complex a variable to be used in a simple linear or dichotomized fashion [3–6]. Spline functions or fractional polynomial regression models may be preferable in such settings. As an aside, matching individuals on BMI might have enabled the investigators to tease out any independent effects of T2DM.

Numerically, it may seem as if the presence of overweight reduced GLS to a greater extent among patients with T2DM as compared with non-diabetics, and this is certainly supported by the Cardiovascular Continuum concept, by which hyperglycemia accelerates the cardiovascular aging process [7]. However, the potential for a type 2 error should not be overlooked. Assuming a 1:1 ratio of overweight versus non-overweight healthy controls, a sample size of 270 would have been sufficient to render a significant two-sided P value for the observed GLS difference of 0.6, with 80% power. With the same power, 200 patients with T2DM would be required to show statistical significance at a difference of 1.0. This might explain why only a few echocardiographic variables reached statistically significant differences in Table 2 in the original paper [1]. Accordingly, we encourage the authors to provide the results of an appropriate interaction analysis to better clarify this issue.

Finally, in the previous study, BMI was independently associated with incident echocardiographic abnormalities at the expense of insulin sensitivity [2]. The frequent co-occurrence of insulin resistance, hyperinsulinemia, obesity, hypertension, and T2DM makes it difficult to dissect the separate role of each of these conditions for the development of subclinical cardiac damage, and studies of insulin and cardiac structure and function have revealed inconsistent findings [8–10]. Therefore, we believe the authors have a golden opportunity to examine associations between insulin and the most sensitive marker of impaired LV function, GLS.

## Abbreviations

BMI: body mass index
EF: ejection fraction
GLS: global longitudinal strain
LV: left ventricle/left ventricular
LVEF: left ventricular ejection fraction
T2DM: type 2 diabetes mellitus
